# Evaluation of C-Reactive Protein, Endothelin-1, Adhesion Molecule(s), and Lipids as Inflammatory Markers in Type 2 Diabetes Mellitus Patients

**DOI:** 10.1155/2007/73635

**Published:** 2007-02-25

**Authors:** Hala El-Mesallamy, Salwa Suwailem, Nadia Hamdy

**Affiliations:** ^1^Biochemistry Department, Faculty of Pharmacy, Ain Shams University, Abbassia, Cairo 11566, Egypt; ^2^Cardiology Department, Faculty of Medicine, Ain Shams University, Abbassia, Cairo 11566, Egypt

## Abstract

This study compared lipids, the product of lipid peroxidation malondialdehyde (MDA), the acute phase reactant high-sensitive C-reactive protein (hsCRP), endothelin-1 (ET-1), *P*-selectin, intercellular adhesion molecule-1 (ICAM-1), and vascular cell adhesion molecule-1 (VCAM-1) between healthy controls, subjects with ischemic heart disease (IHD) and type 2 diabetes mellitus (DM) subjects who did not perform coronary artery bypass graft (CABG) surgery as well as type 2 DM subjects who performed CABG. HbA_1c_, lipids, MDA, hsCRP, ET-1, *P*-selectin, ICAM-1, and VCAM-1 levels were significantly higher in the diabetic groups than in either healthy controls or IHD subjects. In the diabetic groups, there was a negative association among hsCRP and HDL-C. ET-1, ICAM-1 levels, and TAG were positively correlated, as do the association between *P*-selectin, VCAM-1, and HbA_1c_%. Also a positive relation was found among hsCRP levels and ICAM-1, as well as MDA and ET-1. *P*-selectin and ICAM-1 were significantly positively correlated. This study indicates that increased level of oxidative stress marker, proinflammatory markers, and their downstream effectors adhesion molecules occur in type 2 DM.

## 1. INTRODUCTION

Cardiovascular (CV) morbidity is a major burden in patients with type 2 diabetes mellitus (type 2 DM), with endothelial dysfunction as an early sign of diabetic vascular disease [1] that is related to the presence of a vascular low-grade inflammation. Alteration in endothelin-1 (ET-1) balance of the endothelium is the key event in the initiation of arteriosclerosis [2], via activation of leukocyte adhesion [3], which is linked to the presence of a vascular inflammation. Cellular adhesion molecules (CAMs), namely intercellular adhesion molecule-1 (ICAM-1), and vascular cell adhesion molecule-1 (VCAM-1), are poorly expressed by the resting endothelium, but are upregulated during inflammatory atherogenesis and may be an index of endothelial activation or even a molecular marker of early atherosclerosis [4]. Postprandial rises in hyperglycemia can trigger endothelial damage through increased oxidative stress [5]. Also, type 2 DM is associated with increased risk for complications following coronary artery bypass grafting (CABG) surgery, by inducing inflammatory vascular dysfunction [6].

Therefore, this study aimed at measuring the levels of lipids, the lipid peroxidation product malondialdehyde (MDA), acute-phase reactant high-sensitive C-reactive protein (hsCRP), ET-1, *P*-selectin, ICAM-1, and VCAM-1 among healthy controls and ischemic heart disease (IHD) subjects, type 2 DM subjects who did not perform CABG surgery, as well as type 2 DM subjects who performed CABG. In addition to evaluating if ET-1 and CAMs may be upregulated during inflammatory atherogenesis, an index of inflammatory endothelial activation may be better than assessment of the conventional CV risk factors. Moreover, the present work primarily aimed to study the interactions between type 2 DM and susceptibility of lipoproteins to oxidation, and their relation to inflammation. Since it is well known that type 2 DM subjects have multiple risk factors that potentiate each other, we limit our study to nonsmoker male diabetic individuals.

## 2. SUBJECTS AND METHODS

### 2.1. Subjects

The studied groups included (57) males, of which (12) males served as healthy controls (group I). The control group was selected from subjects that attended the outpatient endocrine clinic at Ain Shams University Specialized Hospitals (ASUSH). None of the healthy controls, take any medication or dietary supplements including vitamin(s) and/or antioxidant(s). Forty five males were selected from patients admitting to ASUSH Cardiology Department. After protocol approval, the study was conducted in the period from September 2004 to May 2005. Patients in the study were classified into the following groups: ischemic heart disease subjects without DM (group II) (*n* = 15), type 2 DM subjects who did not perform CABG (group III) (*n* = 15), and type 2 DM subjects who performed CABG surgery not less than one year (group IV) (*n* = 15). All subjects gave written informed consent prior to participation and the study was approved by the Committees on Medical Ethics of the ASUSH. The study was carried out in accordance with the regulations and recommendations of the Declaration of Helsinki. A detailed medical history and drug treatment(s) were collected for all subjects. The following exclusion criteria were used for all subjects: age more than 65 or less than 40 years, acute and chronic inflammatory diseases, liver disease, macroalbuminuria, not having recently received any anti-inflammatory drugs. After analysis of either diabetic or nondiabetic people, if each one fulfilled the criteria to participate either in the control or in the diabetic groups, an invitation was given by one of the authors. Hypertension was defined as history of arterial hypertension with or without antihypertensive treatment and/or > 130 mm Hg systolic and/or > 80 mm Hg diastolic arterial blood pressure (mean of 3–5 repeated blood pressure measurements). BMI was calculated as an index of the weight in kilograms divided by the square of the height in meters. All subjects were asked to discontinue taking aspirin, if they were using it, at least two weeks prior to blood collections. Inclusion criteria include type 2 DM of 10–15 years duration, criteria for type 2 DM patients include age at diagnosis > 35 years.

### 2.2. Laboratory procedures

All subjects were advised to take no medication on the morning before blood sample collection. Initially, fasting blood samples (5–10 mL) were taken between 8 : 00 and 10 : 00 a.m. Blood was obtained from the anticubital vein, after an overnight fasting period. Samples were divided into two parts; one containing Na_2_-EDTA (final concentration 1 mg/mL) for glycosylated hemoglobin HbA_1c_% determination by Stanbio Glycohemoglobin (USA) [7]. The other part was taken into vacutainer clotted tubes, where sera were obtained by centrifugation at 3000 rpm at 4°C for 10 minutes. Sera were separated, aliquoted first for the measurement of fasting blood glucose (FBG) [8] and lipids (total cholesterol (TC) [9] and triacylglycerol (TAG) [10]) by using standard enzymatic techniques. HDL-C was determined after precipitation of apolipoprotein B-containing lipoproteins [11], and finally LDL-C was calculated according to the Friedwald formula (FF) : LDL-C = TC-TAG/5 (mg/dL) [12]. The reference values for the lipid profile were according to established guidelines [13].

### 2.3. Analytical determinations

#### 2.3.1. MDA determinations

Oxidative susceptibility of LDL was measured as the level of MDA [14].

#### 2.3.2. CRP, ET-1, and CAMs determinations

Serum aliquots were kept frozen at −70°C for hsCRP, ET-1, *P*-selectin, ICAM-1, and VCAM-1. ELISA procedures were carried out according to the manufactures instructions.

### 2.4. Statistical analysis

All statistical analyses were performed using SPSS version 9 software package. Data are presented as mean ± SD if normally distributed, and as median (with the 25th and 75th centiles-quartiles) if not normally distributed (FBG, MDA, hsCRP, ET-1, and CAMs). To determine differences between groups, analysis of variance (ANOVA) followed by Bonferroni's post-hoc analysis was used for multiple comparisons between different groups. When comparing normally distributed variables between patients and healthy controls, an independent *t* test was used for comparing means. For comparison of skewedly distributed variables between the study groups, median values were calculated and Mann-Whitney *U* test was used. The Kruskall-Wallis test for multiple independent samples was used for comparison between the subgroups of patients. Correlations between type 2 DM, CV risk factors, and CRP were evaluated by Spearman's rank correlation. Multiple linear regressions including all participants were used to evaluate whether established type 2 DM and CV risk factors independently predicted the levels of CRP, ET-1, and CAMs. Type 2 DM groups were also introduced into this model together with age, BMI, HbA_1c_, hypertension, and lipids to evaluate if the observed differences in CRP, ET-1, CAMs between type 2 DM patients and healthy controls were independent of the influence of CV risk factors. The level of statistical significance was set at *P* ≤ .05.

## 3. RESULTS

The baseline characteristics of the studied participants are presented in Table 1. The groups did not differ in relation to sex, smoking, and nutritional status as indirectly evaluated by blood (red and white) cell analysis (not shown). Type 2 DM patients (groups III and IV) were overweight, whereas the control group presented normal values (*P* < .01). Inasmuch, HbA_1c_ levels in type 2 DM patients were higher than either those in the control group or the IHD group (*P* < .01). FBG mg/dL levels increased significantly in the diabetic groups III, IV when compared with either the control volunteer or the ischemic group (Table 1). Other characteristics of the groups are depicted in Table 1 (age, systolic, and diastolic blood pressure). Lipids profile (TAG, TC, and LDL-C levels, as well as the CV risk ratio TC/HDL-C) in the different studied groups (ANOVA, Table 1) were significantly increased in groups II, III, and IV when compared with the control group I, except for HDL-C which was decreased in these groups compared with the control group I.

As shown in Table 2, the median values of MDA, hsCRP, ET-1, ICAM-1, and VCAM-1 concentrations were significantly higher in the IHD group and the diabetic groups (groups III, IV) in comparison to those levels obtained in the control group (*P* ≤ .05). On the other hand, no significant differences among the diabetic groups (groups III, IV) were observed in all parameters.

After adjustments for established CV risk factors, being a type 2 DM patient (group III: type 2 DM who did not perform CABG and group IV: type 2 DM who performed CABG) was still an independent statistically significant predictive factor for CRP, ET-1, and *P*-selectin levels (*P* ≤ .05), but not for MDA, ICAM-1, and VCAM-1, where hsCRP, ET-1, and *P*-selectin levels were all dependent on MDA levels (*P* ≤ .05). Also, *P*-selectin was dependent on HbA_1c_%.

**Figure 1:**
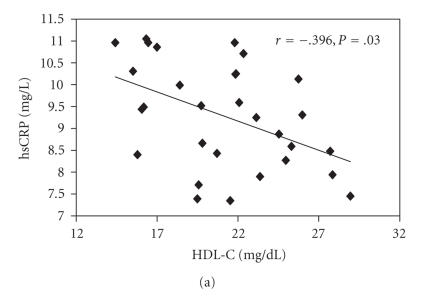

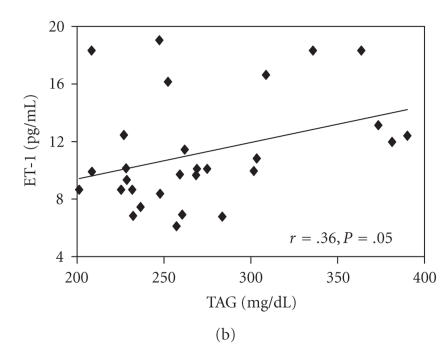

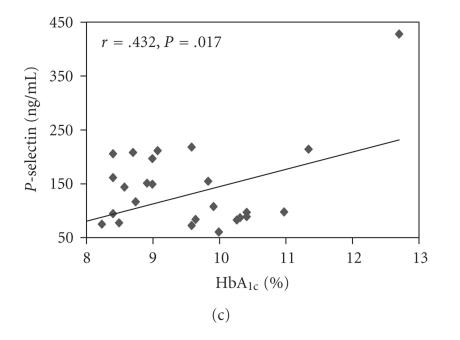

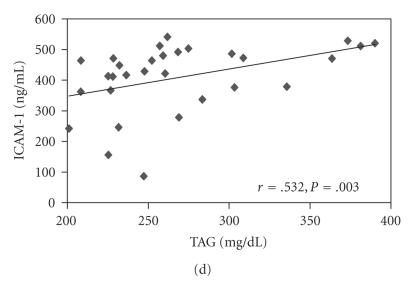

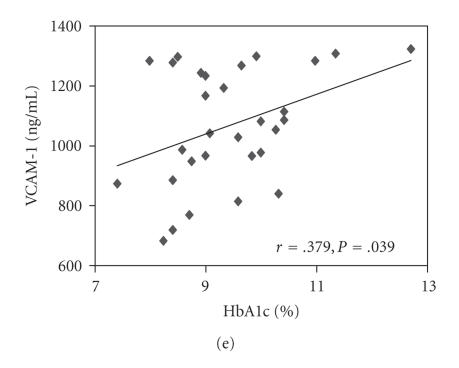
Correlation between (a) hsCRP (mg/L) and HDL-C (mg/dL), (b) ET-1 (pg/mL) and TAG (mg/dL), (c) *P*-selectin (ng/mL) and HbA_1c_(%), (d) ICAM-1 (ng/mL) and TAG (mg/dL), and (e) VCAM-1 (ng/mL) and HbA_1c_(%) in type 2 DM patients (groups III and IV) (*n* = 30). Each individual value is represented by a symbol (▪), *r* = Spearman's rank correlation coefficients.

**Figure 2:**
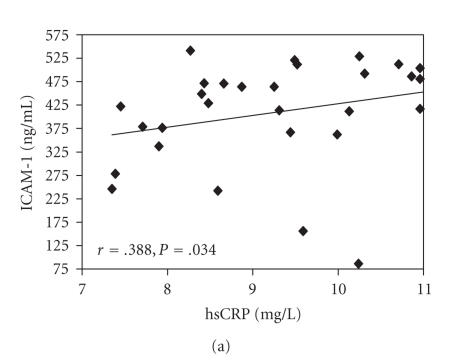

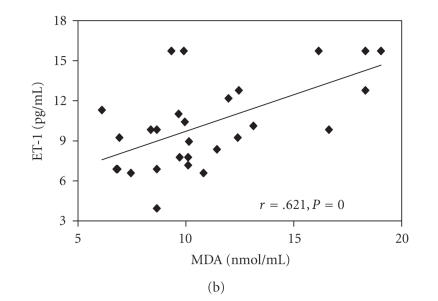

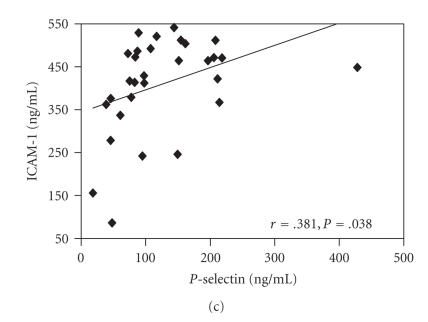
Correlation between (a) MDA (nmol/mL) and ET-1 (pg/mL), (b) hsCRP (mg/L) and ICAM-1 (ng/mL), (c) *P*-selectin (ng/ml) and ICAM-1 (ng/mL) in type 2 DM patients (groups III and IV) (*n* = 30). Each individual value is represented by a symbol (▪), *r* = Spearman's rank correlation coefficients.

In the diabetic groups (groups III, IV) (*n* = 30), a negative association was found among hsCRP levels and HDL-C concentrations (Figure 1(a)). ET-1 levels and TAG concentrations were positively correlated, as did the relation between *P*-selectin and HbA_1c_%, ICAM-1 and TAG, and the association between VCAM-1 and HbA_1c_ (Figures 1(b), 1(c), 1(d), and 1(e)). Also in the diabetic groups (groups III, IV) (*n* = 30), a positive association was observed among MDA values and ET-1 determinations and among hsCRP levels and ICAM-1 determinations (Figures 2(a) and 2(b)). *P*-selectin and ICAM-1 were significantly positively correlated (Figure 2(c)).

## 4. DISCUSSION

Data in this study demonstrate that MDA, hsCRP, Lipids (TAG, TC, and LDL-C), ET-1, and CAMs increased in type 2 DM patients, together with decreased HDL-C levels. These results confirm reports in the literature that a low-grade inflammation exists in type 2 diabetic patients. Atherosclerosis is a multifactorial disease and evidence indicates that certain synergistic risk factors accelerate atherogenesis. When raised TAG coexists with an atherogenic cholesterol profile, the overall risk is enhanced [15]. Impairment of vascular endothelial function is an initial step in the development of inflammatory atherosclerosis [16].

The mechanism of the elevation of CAMs may be contributed to hyperglycemia, hyperinsulinemia, oxidative stress, inflammation, and insulin resistance [17]. Endothelial dysfunction, as it relates to both ET-1 and CAMs, might be considered not at the level of EC activation, leukocyte aggregation, and activation, but rather at the point where inflammatory oxidative stress is increased. The earliest event following plaque fissure is the adhesion and aggregation of platelets leading to thrombus formation. Increased platelet aggregation, in response to inflammation, contributes to the development of atherosclerosis and increases the risk of myocardial infarction [18]. In our study, the level of MDA in serum was found to be higher in diabetic patients (groups III, IV) than in control subjects (groups I, II) as well as being associated with lipids and ET-1 positively. This confirms that in atherosclerotic plaque activated macrophages and neutrophils release several kinds of oxidants, which in high concentrations lead to oxidative stress causing damage to lipids. Furthermore, the findings of raised levels of hsCRP in diabetic patients are in accordance with that the inflammatory course of the atherosclerotic process is more severe in diabetic patients than in nondiabetic subjects [19]. Taken together, these findings suggest that these groups of diabetic patients present a low-grade inflammation triggered by the diabetic mellitus state, which is favoring the progression of accelerated atherosclerosis. In agreement with this, our study found that hsCRP is not a surrogate marker for CVD in diabetic patients. However, further studies are needed to better evaluate the outcome of the diabetic subgroup that presented high levels of CRP. Recently, Schulze et al. [20] reported that high plasma levels of CRP were associated with an increased risk of CV events among diabetic men, independent of currently established lifestyle risk factors, blood lipids, and glycaemic control [20]. Regarding the present study, one cannot rule out the possibility that the presence of obesity, significant hyperglycaemia, and the low levels of HDL-cholesterol in the diabetic group may have contributed to these results. In addition, the finding that serum levels of both CRP and HDL-C are negatively correlated provides a further indication that both variables contribute to the vascular inflammatory process. Several CV disorders, including atherosclerosis, are associated with endothelial dysfunction [21], as well as enhanced expression of CRP and ET-1. The higher incidence of atherosclerotic vascular disease in patients with type 2 DM may also be related to the atherogenic properties of ET-1 [3]. *P*-selectin level tended to be higher in type 2 DM subjects compared with groups I and II, but this difference did not achieve statistical significance. This result may be explained by the different patterns of CAMs expression in various cell types and tissues. Activated platelets play an important role in coagulation and release of *P*-selectin [22]. ICAM-1 is mostly expressed in endothelial cells (ECs) and their expression is enhanced by a variety of proinflamatory stimuli [23].

Diabetes is associated with increased risk for complications following CABG surgery, as the vasoconstrictor ET-1 is elevated in diabetic patients, following CABG [6]. Tissue damage after ischemia reperfusion involves leukocyte endothelial interactions mediated by CAMs [24]. Augmented superoxide production plays an important role in diabetic complications by causing vascular dysfunction. A recent study showed that platelets from patients after CABG show an increased expression of *P*-selectin, a marker of *α*-granule secretion associated with the progression of atherosclerosis [25]. Accordingly, our study investigated ET-1 and CAMs levels in type 2 DM patients who performed CABG (group IV). Hyperlipidemia is an important pathogenic mechanism of inflammatory endothelial dysfunction in patients with type 2 DM. In this study, lipids profile, MDA, CRP, ET-1, and CAMs levels were elevated, and correlated in type 2 DM patients who performed or did not perform CABG. Hence, increased endothelial activity, in this study is related and correlated to hyperglycemia, dyslipidemia, and inflammation observed in these groups, suggesting that type 2 DM-associated metabolic disorders can explain the significant impairment of endothelial function and CAMs. Spearman's correlations showed that *P*-selectin and VCAM-1 were significantly positively correlated to HbA_1c_, while ET-1 and ICAM-1 were correlated to TAG in groups III and IV (*n* = 30). Also *P*-selectin was correlated positively significantly with ICAM-1. The direct correlation with HbA_1c_ suggested that elevated glucose concentrations were responsible for endothelial activation and that hyperglycemia increases CAMs, which reflects excessive formation of atherosclerotic plaques in patients with disturbances of glucose metabolism [26]. The direct relationship with plasma lipoproteins could suggest that hyperdyslipidemia affects CAMs secretion in vivo. Thus CRP, ET-1, *P*-selectin, ICAM-1, and VCAM-1 levels are related to both hyperglycemia and dyslipidemia and may reflect the presence of a multiple-risk factor clustering syndrome providing further support for the role of these markers in atherosclerosis. Taken together, these data strongly suggest that type 2 DM-associated metabolic disorders can explain the significant impairment of endothelial function and CAMs.

## 5. CONCLUSIONS

Our study shows that hsCRP, ET-1, and CAMs as surrogate markers of CV inflammation are elevated in diabetic patients. Taken together, these data support the opinion that diabetic patients present a high risk for CVD and need early aggressive intervention. Increased oxidative stress and inflammation in type 2 DM could be partly overcome by antioxidant administration.

## Figures and Tables

**Table 1:** Clinical and laboratory characteristics of the studied groups; healthy controls (group I), ischemic heart disease (group II), type 2 DM who did not perform CABG (group III), and type 2 DM who performed CABG (group IV). Data are mean ± SD. *n* = number of subjects, SBP = systolic blood pressure, DBP = diastolic blood pressure, BMI = body mass index, FBG = fasting blood glucose.

Parameters	Group I	Group II	Group III	Group IV

*n*	12	15	15	15

Age (years)	45 ± 1	53 ± 5^(a)^	52 ± 4^(a)^	55 ± 5^(a)^
SBP (mm Hg)	104 ± 5	129 ± 17^(a)^	143 ± 12^(a, b)^	147 ± 13^(a, b)^
DBP (mm Hg)	73 ± 6	86 ± 11^(a)^	93 ± 5^(a)^	92 ± 7^(a)^
BMI (Kg/m^2^)	24 ± 1	25 ± 1^(a)^	32 ± 1^(a, b)^	31 ± 3^(a, b)^
FBG (mg/dL) §§	106 (99–114)	108 (80–140)	255 (225–277)^(a, b)^	229 (220–249)^(a, b)^
HbA_1c_(%)	6 ± 1	6 ± 1	9 ± 1^(a, b)^	9 ± 1^(a, b)^
TAG (mg/dL)	141 ± 26	204 ± 27^(a)^	256 ± 45^(a, b)^	283 ± 59^(a, b)^
TC (mg/dL)	201 ± 6	242 ± 13^(a)^	405 ± 53^(a, b)^	373 ± 53^(a, b)^
HDL-C (mg/dL)	45 ± 3	32 ± 3^(a)^	22 ± 4^(a, b)^	21 ± 5^(a, b)^
LDL-C (mg/dL)	127 ± 11	169 ± 16^(a)^	332 ± 51^(a, b)^	296 ± 56^(a, b)^
TC/HDL-C	4 ± 0.3	8 ± 1^(a)^	19 ± 3^(a, b)^	19 ± 4^(a, b)^

§§ = median (with the 25th and 75th centiles-quartiles).

^(a, b, c)^ Significant difference from healthy controls, IHD group, type 2 DM group who did not perform CABG, respectively, at *P* ≤ .01.

**Table 2:** Serum concentrations of the studied parameters in healthy controls (group I), ischemic heart disease (group II), type 2 DM who did not perform CABG (group III), and type 2 DM who performed CABG (group IV). Values are median (with the 25th and 75th centiles-quartiles).

Groups/parameters	Group I	Group II	Group III	Group IV

*n*	12	15	15	15

MDA (nmol/mL)	1 (0-1)	4 (1–4)^(a)^	9 (7–13)^(a, b)^	10 (9–12)^(a, b)^
hsCRP (mg/L)	0.4 (0-1)	3 (2–4)^(a)^	9 (8–10)^(a, b)^	10 (9–11)^(a, b)^
ET-1 (pg/mL)	3 (1–3)	2 (2–4)	10 (9–18)^(a, b)^	10 (9–12)^(a, b)^
*P*-selectin (ng/mL)	67 (60–110)	52 (14–137)	95 (48–212)^(a)^	98 (84–155)
ICAM-1 (ng/mL)	140 (107–194)	236 (153–284)^(a)^	367 (246–449)^(a, b)^	473 (429–512)^(a, b)^
VCAM-1 (ng/mL)	608 (388–848)	625 (247–944)	1167 (987–1284)^(a, b)^	977 (840–1244)^(a, b)^

(a, b, c) Significant difference from healthy controls, IHD group, type 2 DM group who did not perform CABG, respectively, at *P* ≤ .05.
